# MERTK as a novel therapeutic target in head and neck cancer

**DOI:** 10.18632/oncotarget.8724

**Published:** 2016-04-13

**Authors:** Anne von Mässenhausen, Christine Sanders, Britta Thewes, Mario Deng, Angela Queisser, Wenzel Vogel, Glen Kristiansen, Stefan Duensing, Andreas Schröck, Friedrich Bootz, Peter Brossart, Jutta Kirfel, Lynn Heasley, Johannes Brägelmann, Sven Perner

**Affiliations:** ^1^ Section of Prostate Cancer Research, University Hospital of Bonn, Bonn, Germany; ^2^ Institute of Pathology, University Hospital of Bonn, Bonn, Germany; ^3^ Center for Integrated Oncology Cologne/Bonn, University Hospital of Bonn, Bonn, Germany; ^4^ Pathology of the University Hospital of Luebeck, Luebeck, Germany; ^5^ Leibniz Research Center Borstel, Borstel, Germany; ^6^ Department of Urology, University of Heidelberg, Heidelberg, Germany; ^7^ Department of Otorhinolaryngology/Head and Neck Surgery, University Hospital of Bonn, Bonn, Germany; ^8^ Department of Hematology/Oncology, University Hospital of Bonn, Bonn, Germany; ^9^ Department of Craniofacial Biology, University of Colorado Anschutz Medical Campus, Aurora, Colorado, USA

**Keywords:** head and neck cancer, MERTK, targeted therapy

## Abstract

Although head and neck cancer (HNSCC) is the sixth most common tumor entity worldwide therapy options remain limited leading to 5-year survival rates of only 50 %. *MERTK* is a promising therapeutic target in several tumor entities, however, its role in HNSCC has not been described yet. The aim of our study was to investigate the biological significance of MERTK and to evaluate its potential as a novel therapeutic target in this dismal tumor entity. In two large HNSCC cohorts (n=537 and n=520) we found that MERTK is overexpressed in one third of patients. *In-vitro,* MERTK overexpression led to increased proliferation, migration and invasion whereas MERTK inhibition with the small molecule inhibitor UNC1062 or MERTK knockdown reduced cell motility via the small GTPase RhoA.

Taken together, we are the first to show that MERTK is frequently overexpressed in HNSCC and plays an important role in tumor cell motility. It might therefore be a potential target for selected patients suffering from this dismal tumor entity.

## INTRODUCTION

Head and neck squamous cell carcinoma (HNSCC), which represents more than 95 % of head and neck cancers, is the sixth most common tumor entity worldwide. Therapy options are mostly restricted to surgery and radio- and/or chemotherapy resulting in a 5-year survival rate of around only 50 % depending on the stage at time of diagnosis [1, 2].

Several risk factors such as smoking and alcohol consumption as well as human papilloma virus (HPV) infection have been identified [3–5]. However, an improved knowledge of the biological mechanisms leading to tumorigenesis and tumor progression is key to develop successful targeted therapies that improve survival and reduce toxicities associated with current non-selective treatment strategies.

Receptor tyrosine kinases (RTKs) are promising targets for cancer and other diseases and several drugs have been developed and are already FDA approved [6]. Different RTKs have been implicated in HNSCC. Epidermal growth factor receptor (*EGFR*) amplification and expression correlate with poor survival in HNSCC [7–9]. Accordingly several clinical trials could show beneficial effects for patients when EGFR was targeted with cetuximab [10, 11]. However, neither EGFR expression nor *EGFR* copy-number are predictive of response to this therapy and other EGFR-targeting agents such as panitumumab or erlotinib failed to significantly improve survival rates [10, 12–15]. Other RTKs that are currently investigated in clinical trials for patients with HNSCC include the insulin-like growth factor 1 receptor (IGF1R) [16] and the MET proto-oncogene receptor tyrosine kinase (MET) in combination with the vascular endothelial growth factor receptor (VEGFR) [17]. Despite the involvement of these RTKs in HNSCC the identification of novel therapeutic approaches in HNSCC remains crucial especially with regard to potential resistance mechanisms that have already been described for EGFR targeted therapy in HNSCC [18].

MER proto-oncogene tyrosine kinase (*MERTK*) belongs to the family of TAM RTKs [19] and is physiologically expressed in cells of the hematopoietic lineages such as macrophages, dendritic cells and natural killer cells [20]. Moreover, its up-regulation has been shown in different cancer entities: 69 % of non-small cell lung cancers overexpress MERTK where it is involved in tumor growth and chemosensitivity [21]. Moreover, MERTK has been described as a potential therapeutic target in melanoma [22], astrocytoma [23] as well as gastric [24] and prostate cancer [25]. Depending on the cell type and tissue microenvironment receptor activation can lead to higher cell proliferation by signaling via the PI3K/AKT, ERK1/2, BCL-2 and NFκB pathways and to increased migration of cells through signaling via the focal adhesion kinase (FAK) and RhoA [26].

Due to the growing evidence of MERTK's involvement in different cancers several selective small molecule inhibitors have recently been developed and show promising pre-clinical results [27]. One of them is UNC1062, which inhibits MERTK phosphorylation and colony formation in different tumor cell lines [22, 24, 28]. Moreover, UNC1062 induced apoptosis and reduced MERTK-mediated downstream signaling as well as invasion in melanoma cells [22] and proliferation in gastric cancer cell lines [24].

To date nothing is known about the role of MERTK in HNSCC. In this study we are the first to show that MERTK is overexpressed in around one third of patients and provide evidence for an oncogenic role of MERTK in HNSCC. *In-vitro,* its overexpression increased cell motility whereas suppression of MERTK signaling pathways using UNC1062 or shRNA led to reduced migration and invasion without altering cell survival. Taken together these results establish MERTK as a novel therapeutic target in HNSCC.

## RESULTS

### Analysis of MERTK expression in patients with HNSCC

**Figure 1:**
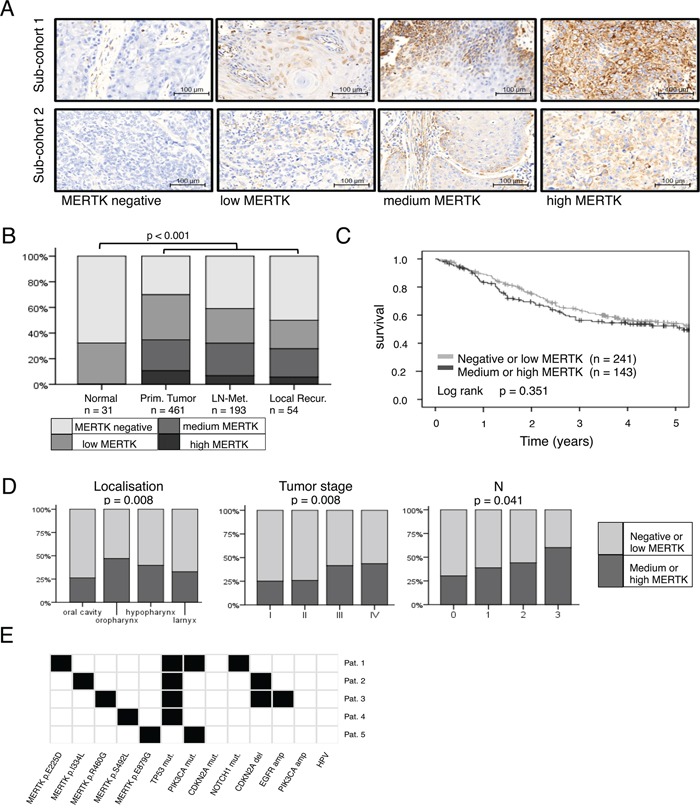
MERTK expression is increased in head and neck cancer **A.** Representative cores from primary tumors for each MERTK staining intensity for both staining protocols/sub-cohorts. **B.** Protein expression of MERTK in normal mucosa (n = 31), primary tumors (n = 461), lymph node metastases (n = 193) and local recurrences (n = 54). **C.** Kaplan-Meier estimates for overall survival of patients with negative or low MERTK protein expression compared to patients with medium or high expression. **D.** MERTK protein expression in different primary tumor localizations, tumor- and N-stages. **E.** MERTK mutations in HNSCC (n = 5/279) and additional mutations found in these five patients. (B: Fisher test: Monte Carlo, 100 000 random samples; C: log-rank test; D: Fisher test: Monte Carlo, 100 000 random samples).

In total, we quantified MERTK protein expression in 739 tissue samples (31 normal mucosa, 461 primary tumors, 193 lymph node metastases and 54 locally recurrent tumors) of 537 patients by immunohistochemistry (IHC) in the Bonn HNSCC cohort. Clinical information was available for 449 of these patients (83.6 %, Table 1). Due to variable staining intensities of two different lot numbers of the antibody for two batches of TMA slides MERTK expression was first classified into four categories from negative to low, medium and high MERTK expression within each of these sub-cohorts (Figure 1A). However, as the samples in both sub-cohorts differed only in their anatomic localization of the primary tumor and the patient's sex (Supplementary Tables S1 and S2), results were pooled for further analysis. In the majority of normal mucosa samples (67.7 %) MERTK expression was completely absent and low in the remaining cases. Cases with medium or high MERTK expression were significantly enriched in all types of tumor tissue compared to normal mucosa (p < 0.001). Because normal mucosa showed either no or low MERTK expression, we considered these expression levels as physiological and medium or high expression of MERTK as overexpression. With this definition in total 34.7 % of primary tumors, 32.2 % of lymph node metastases and 27.8 % of recurrences but 0 % of normal samples showed MERTK overexpression (Figure 1B). To validate our findings in an independent cohort we analyzed MERTK mRNA expression data available from The Cancer Genome Atlas (TCGA). In the TCGA cohort there also was a trend towards higher MERTK mRNA levels with 29 % of primary tumor samples having an expression above the 85^th^ quantile as compared to normal mucosa (p = 0.16, Supplementary Table S4). In the univariate analysis no association of MERTK protein expression with prognosis was evident (Log rank p = 0.351) with 5-year survival rates of 51.2 % and 49.4 % for patients with no/low or medium/high MERTK protein expression, respectively for the Bonn HNSCC cohort (Figure 1C). When adjusting for age, tumor stage, HPV, alcohol abuse and smoking the Cox regression model showed no significantly increased hazard ratio for patients with medium/high MERTK expression (p = 0.327, Hazard ratio = 1.215, Supplementary Table S3). However, we observed lower MERTK protein levels in tumors from the oral cavity compared to pharynx and larynx carcinomas in the Bonn HNSCC cohort (p = 0.008, Figure 1D, Table 1). The higher MERTK expression in tumors outside the oral cavity could be confirmed in the TCGA HNSCC cohort by assessing MERTK mRNA expression levels (p < 0.001, Supplementary Table S4). Moreover, in the Bonn HNSCC cohort, MERTK expression increased with higher tumor stages (p = 0.008) and more advanced regional lymph node status (p = 0.041, Figure 1D Table 1). Both findings could be validated in the TCGA HNSCC cohort (p = 0.046 and p = 0.011, respectively; Supplementary Table S4). Additionally, in the TCGA HNSCC cohort higher tumor grading correlated with higher MERTK mRNA (p < 0.001, Supplementary Table S4) and for the Bonn HNSCC cohort the same trend was evident (p = 0.246, Table 1, Jonckheere trend test p = 0.094). In the Bonn HNSCC cohort, no correlation was found between MERTK protein expression and sex, age, alcohol consumption, smoking habits or HPV status (Table 1). In contrast, in the TCGA HNSCC cohort MERTK mRNA expression levels were higher in smokers (p = 0.034) and HPV-positive patients (p < 0.001, Supplementary Table S4).

**Table 1:** MERTK expression and clinico-pathological features of the Bonn HNSCC cohort

	Bonn HNSCC cohort						
	Total number of patients n=537		MERTK negative	Low MERTK	Medium MERTK	High MERTK	p-value
**Tissues Available**							
Normal	31 (19*)		21 (67.7 %)	10 (32.3 %)	0 (0.0 %)	0 (0.0 %)	normal mucosa vs. tumor tissue **< 0.001**^(2)^
Primary tumor	461 (52*)		139 (30.2 %)	162 (35.1 %)	111 (24.1 %)	49 (10.6 %)	
Lymph node metastasis	193 (18*)		79 (40.9 %)	52 (26.9 %)	49 (25.5%)	13 (6.7 %)	
local recurrence	54 (8*)		27 (50.0 %)	12 (22.2 %)	12 (22.2%)	3 (5.6 %)	
	Patients with clinical data n=449	Number of primary tumors	MERTK negative	Low MERTK	Medium MERTK	High MERTK	P-value(no or low vs. medium or high MERTK expression)
**Gender**							
female	338 (75.3 %)	99	28 (28.3 %)	34 (34.3 %)	32 (32.3 %)	5 (5.1 %)	0.905 ^(1)^
male	111 (24.7 %)	310	95 (30.6 %)	102 (32.9 %)	70 (22.6 %)	43 (13.9 %)	
**Age** [mean years, SD]	62.40 (10,87)		62.76 (11.6)	61.43 (10.0)	62.7 (11.6)	62.4 (10.4)	
**Anatomic localiza-tion of Primary**							
Oral Cavity	110 (24.5 %)	99	33 (33.3 %)	40 (40.5 %)	21 (21.1 %)	5 (5.1 %)	**0.008** ^(2)^
Oropharynx	143 (31.8 %)	132	36 (27.3 %)	34 (25.8 %)	42 (31.7 %)	20 (15.2 %)	
Hypopharynx	57 (12.7 %)	53	16 (30.2 %)	16 (30.2 %)	12 (22.6 %)	9 (17.0 %)	
Larynx	131 (29.2 %)	119	36 (30.3 %)	44 (37.0 %)	26 (21.8 %)	13 (10. 9%)	
Unknown	8 (1.8 %)						
**Tobacco**							
Never-Smoker	41 (9.1 %)	35	11 (31.4 %)	10 (28.6 %)	10 (28.6 %)	4 (11.4 %)	0.855 ^(1)^
Ever-Smoker	306 (68.2 %)	278	82 (29.5 %)	90 (32.4 %)	68 (24.4 %)	38 (13.7 %)	
Unknown	102 (22.7 %)						
**Alcohol**							
Non-drinker	122 (27.2 %)	107	33 (30.9 %)	38 (35.5 %)	26 (24.3 %)	10 (9.3 %)	0.144 ^(2)^
Occasional	76 (16.9 %)	71	19 (26.8 %)	27 (38.0 %)	14 (19.7 %)	11 (15.5 %)	
Medium-Heavy	127 (28.3 %)	116	35 (30.2 %)	28 (24.1 %)	35 (30.2 %)	18 (15.5 %)	
Unknown	124 (27.6 %)						
**HPV Status**							
Positive	36 (8.0 %)	35	9 (25.7 %)	9 (25.7 %)	12 (34.3 %)	5 (14.3 %)	0.144 ^(1)^
Negative	413 (92.0 %)	374	114 (30.4 %)	127 (34.0 %)	90 (24.1 %)	43 (11.5 %)	
**T-Stage of Primary**							
T1	116 (25.8 %)	101	29 (28.7 %)	40 (39.6 %)	24 (23.8 %)	8 (7.9 %)	0.229 ^(2)^
T2	149 (33.1 %)	136	43 (31.6 %)	47 (34.5 %)	33 (24.3 %)	13 (9.6 %)	
T3	106 (23.6 %)	97	29 (29.9 %)	27 (27.8 %)	26 (26.8 %)	15 (15.5 %)	
T4	74 (16.4 %)	71	20 (28.2 %)	20 (28.2 %)	19 (26.7 %)	12 (16.9 %)	
Unknown	4 (0.008 %)						
**N Stage of Primary**							
N0	201 (44.8 %)	185	58 (31.4 %)	71 (38.4 %)	38 (20.5 %)	18 (9.7 %)	**0.041** ^(2)^
N1	72 (16.0 %)	67	18 (26.9 %)	23 (34.3 %)	17 (25.4 %)	9 (13.4 %)	
N2	161 (35.9 %)	143	44 (30.7 %)	36 (25.2 %)	44 (30.8 %)	19 (13.3 %)	
N3	6 (1.3 %)	5	1 (20.0 %)	1 (20.0 %)	2 (40.0 %)	1 (20.0 %)	
Unknown	9 (2.0 %)						
**M Stage of Primary**							
M0	432 (96.2 %)	395	117 (29.6 %)	131 (33.2 %)	99 (25.1 %)	48 (12.1 %)	0.389 ^(1)^
M1	16 (3.6 %)	13	6 (46.2 %)	4 (30.8 %)	3 (23.0 %)	0 (0.0 %)	
Unknown	1 (0.2 %)						
**Tumor stage of Primary**							
I	83 (18.5 %)	76	24 (31.6 %)	33 (43.4 %)	14 (18.4 %)	5 (6.6 %)	**0.008** ^(2)^
II	67 (14.9 %)	58	20 (34.5 %)	23 (39.7 %)	11 (19.0 %)	4 (6.9 %)	
III	86 (19.2 %)	82	20 (24.4 %)	28 (34.2 %)	23 (28.0 %)	11 (13.4 %)	
IV	203 (45.2 %)	184	57 (31.0 %)	47 (25.5 %)	53 (28.8 %)	27 (14.7 %)	
Unknown	10 (2.2 %)						
**Grading**							
G1	2 (0.4 %)	2	1 (50.0 %)	1 (50.0 %)	0 (0.0 %)	0 (0.0 %)	0.246 ^(2)^
G2	17 (3.8 %)	15	5 (33.3 %)	7 (46.7 %)	3 (20.0 %)	0 (0.0 %)	
G3	238 (53.0 %)	217	66 (30.4 %)	72 (33.2 %)	57 (26.3 %)	22 (10.1 %)	
G4	113 (25.2 %)	103	29 (28.2 %)	30 (29.1 %)	27 (26.2 %)	17 (16.5 %)	
Unknown	79 (17.6 %)						

Summary of clinico-pathological features of the cohort used for MERTK expression analyses. For some patients tissue for more than one entity (e.g. normal and primary tumor) could be used in IHC (SD, standard deviation;

* number of stained tissue samples for which clinical information was not available). Significance was tested with (1) Fisher test: exact, (2) Fisher test: Monte Carlo, 100 000 random samples.

In summary, these data show that MERTK is frequently overexpressed in HNSCC and might be a potential target for this tumor entity.

### Analysis of MERTK mutations in patients with HNSCC

In a next step we aimed to identify the prevalence of MERTK mutations in HNSCC as a potential mechanism for MERTK overexpression. To this end we analyzed available sequencing data from TCGA (Figure 1E). We identified non-synonymous *MERTK* mutations in 1.8 % (5/279) of cases. All mutations were missense mutations and affected different regions of the MERTK protein. None of the mutations was predicted to have a strong effect on protein function according to a bioinformatic prediction algorithm. All patients showed alterations in one to three genes frequently described as drivers in HNSCC [29, 30] suggesting that *MERTK* mutations are rather passenger mutations in this tumor entity. Moreover, none of the mutations was recurrent in HNSCC or could be found in any other tumor entity. For this reason we decided to focus on wild type MERTK for further experiments.

### Effect of MERTK overexpression

**Figure 2:**
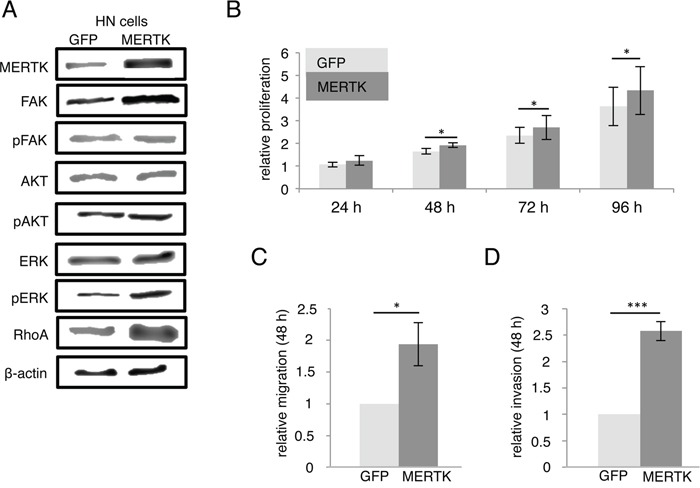
MERTK overexpression increases migration and invasion in HN cells **A.** MERTK overexpression cells compared to GFP control cells with downstream signaling molecules. **B.** Relative proliferation of MERTK overexpression and GFP control cells. **C.** Relative migration of MERTK overexpression and GFP control cells. **D.** Relative invasion of MERTK overexpression and GFP control cells. (B-D: two-tailed paired t-test, n=3, * p < 0.05, *** p < 0.001).

**Figure 3:**
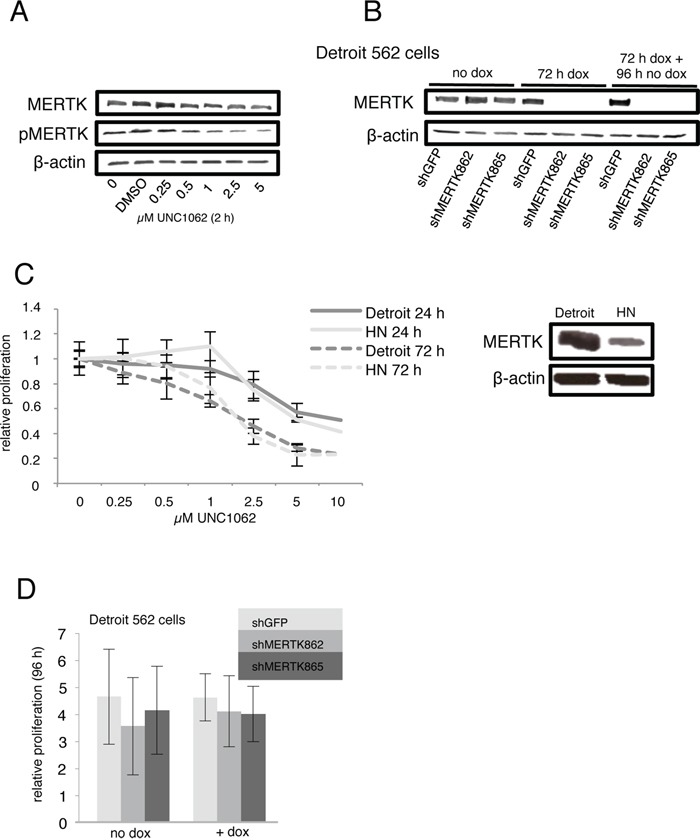
MERTK inhibition with UNC1062 and knockdown does not affect proliferation **A.** Detroit 562 cells were serum-starved for 2 hours and treated with different amounts of UNC1062 for further 2 hours prior to protein extraction. **B.** Knockdown of MERTK in Detroit 562 cells was induced for 72 hours before culturing cells without doxycycline for 4 more days. **C.** Relative proliferation of MERTK high Detroit 562 cells and MERTK low HN cells after treatment with different amounts of UNC1062. **D.** Relative proliferation of Detroit 562 cells with and without induction of MERTK knockdown. (C, D: two-tailed paired t-test, n=3).

To investigate the effect of MERTK overexpression, we stably transfected HN cells, derived from oral squamous cancer [31] with MERTK (Figure 2A). This MERTK overexpression led to a slight, but significant increase in proliferation compared to control cells after 48 hours (p < 0.05) (Figure 2B). Accordingly, an increase in pERK was present by immunoblotting (Figure 3A). The effects on migration and invasion were much more pronounced after 48 hours: MERTK overexpression increased migration and invasion 2-times and 2.5-times, respectively (p < 0.05 and p < 0.001, respectively) (Figures 2C and 2D). Interestingly, immunoblotting of downstream targets involved in cell motility showed an increase in RhoA, but unchanged levels of pFAK, indicating a potential mechanism of action (Figure 2A).

Taken together these data suggest an important role for MERTK in HNSCC especially for migration and invasion of tumor cells.

### Effect of MERTK inhibition and knockdown

To investigate whether MERTK could serve as a therapeutic target in HNSCC we treated Detroit 562 cells, derived from a pleural effusion of a patient with pharynx carcinoma [32], that strongly express MERTK and HN cells with little MERTK expression with UNC1062, a selective MERTK inhibitor [28]. Furthermore, we generated Detroit 562 cells with an inducible MERTK knockdown and respective control cells with shRNA against GFP.

UNC1062 treatment led to a concentration dependent decrease of total as well as phosphorylated MERTK in Detroit 562 after two hours (Figure 3A). MERTK protein levels were successfully abrogated three days after doxycycline induction and remained undetectable for another four days without additional doxycycline treatment (Figure 3B).

Effects of UNC1062 treatment were assessed by evaluation of the viability of treated compared to untreated cells 24 and 72 hours later. For both time points we observed a slightly stronger growth inhibition indicated by less viable cells for Detroit 562 than for HN cells. However, this effect was only observed for low concentrations and did not reach statistical significance (Figure 3C). Knockdown of MERTK did not alter proliferation (Figure 3D).

**Figure 4:**
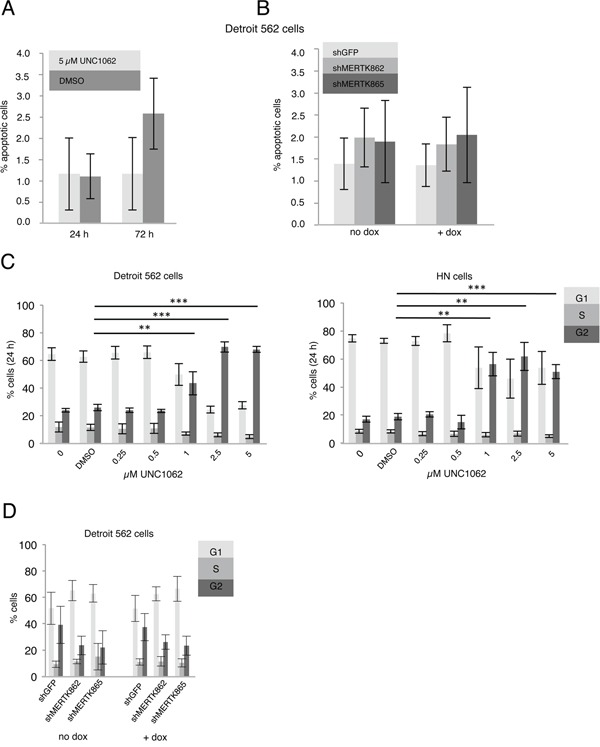
MERTK inhibition with UNC1062 and knockdown has no influence on apoptosis and cell cycle **A.** Detroit 562 cells were treated with 5 μM UNC1062 before determining the number of apoptotic cells. **B.** MERTK knockdown was induced in Detroit 562 cells and number of apoptotic cells was determined. **C.** Detroit 562 and HN cells were treated with increasing amounts of UNC1062 before performing cell cycle analysis. **D.** MERTK knockdown was induced in Detroit 562 cells before performing cell cycle analysis. (A-D: two-tailed paired t-test, n=3, ** p < 0.01, *** p < 0.001).

**Figure 5:**
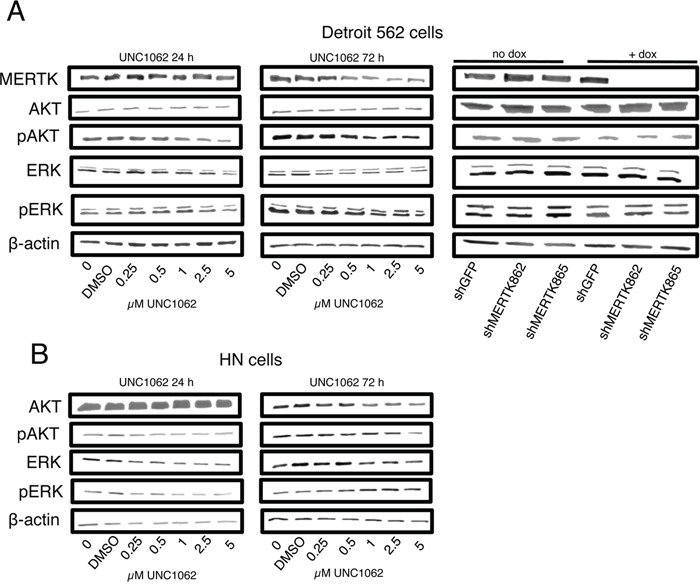
MERTK inhibition with UNC1062 but not MERTK knockdown decreases AKT and ERK phosphorylation **A.** Detroit 562 cells were treated with increasing amounts of UNC1062 or knockdown was induced using doxycycline before isolating proteins. **B.** HN cells were treated with increasing amounts of UNC1062 before isolating proteins. (A, B: n=3).

In flow cytometry analysis neither treatment with UNC1062 nor MERTK knockdown led to an increased apoptosis rate in Detroit 562 cells (Figures 4A and 4B). Interestingly, UNC1062 concentrations above 1 μM induced an arrest in G2 Phase in Detroit 562 as well as HN cells (p < 0.01-0.001 depending on the concentration), whereas MERTK knockdown had no effect on cell cycle (Figures 4C and 4D). Concerning signaling pathways we observed a decreased pAKT and pERK expression after 24 and 72 hours in Detroit 562 but not in HN cells with increasing amounts of UNC1062 (Figures 5A and 5B). However, these pathways were not affected after MERTK knockdown (Figure 5A).

**Figure 6:**
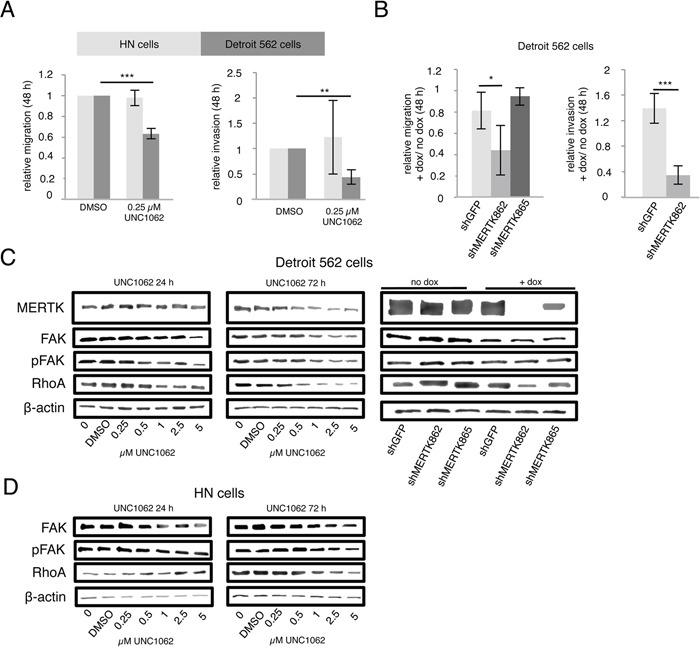
MERTK inhibition with UNC1062 or MERTK knockdown decreases cell migration and invasion via RhoA **A.** Detroit 562 and HN cells were pretreated with 0.25 μM UNC1062 for 24 hours before assessing migration and invasion after further 48 hours. **B.** MERTK knockdown was induced in Detroit 562 cells before assessing migration and invasion after further 48 hours. **C.** Detroit 562 cells were treated with increasing amounts of UNC1062 or knockdown was induced using doxycycline before isolating proteins. **D.** HN cells were treated with increasing amounts of UNC1062 before isolating proteins. (A, B: two-tailed paired t-test, n=3, C, D: n=2, * p < 0.05, ** p < 0.01, *** p < 0.001).

In a next step we aimed to analyze if targeting MERTK with UNC1062 or by knockdown could inhibit migration and invasion. To this end we pretreated Detroit 562 and HN cells with 0.25 μM UNC1062 for 24 hours before assessing migration and invasion. This pretreatment resulted in a 40 % decrease in migration (p < 0.001) and 45 % decrease in invasion (p < 0.01) after UNC1062 treatment in Detroit 562 cells whereas migration and invasion were unaffected in HN cells (Figure 6A). To induce MERTK knockdown in Detroit 562 cells the amount of doxycycline had to be reduced to 0.01 μg/ml since migration was completely abolished even in control cells using higher concentrations. The complete knockdown of MERTK achieved by the shMERTK862 construct led to a significant 60 % decrease in cell migration and invasion (p < 0.05 and p < 0.001, respectively) (Figure 6B). Interestingly, the knockdown with the shMERTK865 construct was less effective after induction with lower doxycycline concentrations and subsequently did not reduce migration. To analyze the pathways involved in MERTK mediated cell motility we determined the expression of the phosphorylated focal adhesion kinase (pFAK) and RhoA. After treating Detroit 562 cells with increasing concentrations of UNC1062 we observed a decrease in pFAK and RhoA expression, which was more pronounced after 72 compared to 24 hours especially for RhoA. In contrast, in HN cells no expression differences could be seen after 24 hours and after 72 hours RhoA and pFAK decreased but only at concentrations above 1 μM (Figures 6C and 6D). Knockdown of MERTK did not influence pFAK expression. However, it led to reduced RhoA levels with stronger effects in the complete compared to the incomplete knockdown (Figure 6C).

In summary our *in-vitro* data suggest that MERTK influences migration and invasion in HNSCC and could be a promising targetable receptor kinase for patients suffering from this dismal disease.

## DISCUSSION

MERTK has been implicated in the tumorgenesis of several cancers such as melanoma, astrocytoma as well as gastric and non-small cell lung cancer [21–24]. However, its role in HNSCC has not been studied, yet. In this report we therefore present data from a comprehensive analysis of the role of MERTK in head and neck cancer. We evaluated MERTK expression in two large independent HNSCC cohorts and its role as a potential therapeutic target by *in-vitro* overexpression, inhibition and knockdown experiments. By analyzing MERTK protein expression we found an overexpression in one third of patients and could show the same trend for mRNA levels. However, protein levels are more significant to predict therapeutic success since the small molecule inhibitors target the protein itself [33]. We found a significant positive correlation between MERTK protein and mRNA expression and clinico-pathological features such as advanced tumor stage and the occurrence of lymph node metastases suggesting that MERTK expression occurs more often in aggressive tumors. Although higher MERTK protein expression was for example found in astrocytoma [23], glioma [34], melanoma [22] as well as non-small cell lung [21], breast [35], and gastric cancer [24], only the last study investigated MERTK expression in context with clinico-pathological data. Schlegel et al. observed higher MERTK expression in metastatic melanoma compared to primary melanoma [22]. These findings could not be confirmed in our HNSCC cohort where MERTK expression is similar in primary tumors compared to lymph node metastases and recurrences. Comparable to our results MERTK overexpression was associated with higher tumor stages in gastric cancer [24], but in gastric cancer patients high MERTK expression was also associated with shorter overall survival. In our HNSCC cohort MERTK was no significant independent predictor for survival after adjustment for clinical covariates. This is most likely due to the fact that MERTK expression is associated with the location of the primary tumor and shows a significant relationship to tumor stage and N status, which are strong determinants for patient survival. In general, parameter estimation in statistical models with co-linear relationships between predictors is less accurate and might prevent significant MERTK effects after adjustment. In addition, it seems plausible that MERTK primarily shows indirect effects on prognosis by promoting tumor cell migration and thereby e.g. increases the number of lymph node metastases and the tumor stage, which in turn lead to worse survival. We therefore suggest that in HNSCC MERTK is important for the metastatic process, which is on the one hand underlined by the fact that patients with high MERTK expression in primary tumors have more often lymph node metastases and on the other hand by our *in-vitro* results, which show a role for MERTK in migration and invasion. In the Bonn as well as in the TCGA HNSCC cohort lower MERTK levels were found in tumors from the oral cavity compared to those from the pharynx and larynx. This fits nicely with the cell models used for our *in-vitro* experiments, since HN cells with almost no MERTK expression are derived from oral-squamous cancer [31] whereas Detroit 562 cells with high MERTK expression were isolated from a pleural effusion from a patient with pharyngeal carcinoma [32].

In the TCGA HNSCC cohort, *MERTK* mutations were present at a very low frequency, were non recurrent and were accompanied by other typical HNSCC mutations. This suggests that *MERTK* mutations most likely are no major drivers in HNSCC. However, focused studies investigating the biological implications of those variants are necessary to fully elucidate the relevance of *MERTK* mutations in HNSCC.

Our *in-vitro* data show that MERTK overexpressing cells proliferated more than GFP control cells. However, this effect was subtle and of little biological relevance. Moreover, knockdown of MERTK did not change proliferation and UNC1062 treatment decreased the number of viable cells independent of MERTK expression level. Nevertheless, we observed a correlation between MERTK and pERK expression. ERK phosphorylation was increased in MERTK overexpressing cells whereas a decrease after UNC1062 treatment was only observed in MERTK high Detroit 562 cells. Similar to our results, in leukemia cells, activation of MERTK led to increased pERK expression without altering proliferation [36]. Also overexpression of MERTK in non-tumorigenic breast cancer epithelial cells did not change proliferation [35]. However, in melanoma and non-small cell lung cancer xenograft mouse models knockdown of MERTK led to impaired proliferation [21, 22]. In gastric cancer UNC1062 treatment decreased proliferation only in MERTK positive cells accompanied by a decrease in ERK phosphorylation. Nonetheless, it remains questionable if the latter is mediated by MERTK inhibition since it was also observed in MERTK negative gastric cell lines [24]. These data suggest that the involvement of MERTK in proliferation and the involved pathways seem to be dependent on the cell type as well as on the microenvironment.

In our cell lines UNC1062 treatment induced a cell cycle arrest in the G2 Phase due to unspecific off-target effects of this inhibitor since we observed this arrest independent of MERTK expression level but not in MERTK knockdown cells. In contrast to results in other tumor entities, there was no correlation between MERTK and apoptosis in HNSCC cells and neither MERTK knockdown nor inhibition with UNC1062 did increase apoptosis rates. Additionally, pAKT as a marker for the PI3K/AKT survival pathway was neither increased in MERTK overexpressing cells, nor down-regulated after MERTK knockdown and only slightly decreased as a result of MERTK inhibition. Studies in melanoma, gastric and lung cancer as well as astrocytoma showed a positive relation between MERTK and pAKT expression and increased apoptosis upon MERTK knockdown or inhibition [21–24]. However, the observed effects differed greatly between the studies suggesting that the influence of MERTK on cell survival and apoptosis vary depending on the cell line and tumor entity.

Finally, we were able to demonstrate that MERTK has a strong influence on cell migration and invasion in HNSCC and that this effect is primarily mediated by RhoA signaling. This was confirmed using overexpression, knockdown and inhibition experiments and is underlined by the fact that higher MERTK protein and mRNA expression correlate with the occurrence of lymph node metastases. Interestingly, migration was only reduced with a complete MERTK knockdown, which was accompanied by decreased RhoA expression. The association of MERTK with cell motility has previously been shown in melanoma, glioblastoma and non-tumorigenic breast epithelial cell lines [22, 35, 37]. In contrast to our results MERTK knockdown in glioblastoma cells decreased migration but increased FAK and RhoA expression [38]. Nevertheless, in HNSCC RhoA was shown to stimulate cell migration. Increased RhoA expression was observed in tumors as compared to normal tissue [39] and *in-vitro* experiments demonstrated that activation of the hyalurone-receptor CD44 promoted cell migration via RhoA in HSC-3 cells [40].

Taken together our *in-vitro* data show that MERTK is a regulator of migration and invasion in HNSCC cells, predominantly modulating cell motility via altered RhoA signaling. To substantiate these results further *in-vitro* studies using additional HNSCC cell lines would be helpful. More importantly, to fully elucidate the biological function of MERTK in metastatic HNSCC and to evaluate its potential as a target, *in-vivo* experiments with e.g. orthotopic mouse models that more closely resemble the tumor microenvironment would be preferable. Orthotopic HNSCC mouse models have the advantage that they are suitable to analyze metastatic behavior of tumor cells [41], but currently UNC1062 is not adapted for mice [42]. To pursue MERTK as a therapeutic target it is therefore important to establish a selective MERTK inhibitor to investigate the *in-vivo* effects of MERTK inhibition.

Our data indicates MERTK to be a potential target in HNSCC that prevents metastatic spread rather than proliferation of main tumor lesions. Therefore we suggest that the inhibition of MERTK could be used in combination with additional antiproliferative therapy, like chemotherapy or the inhibition of an RTK promoting proliferation like EGFR. Apart from the main aim of reducing metastatic spread and reduction of disease progression it is also conceivable that a combination therapy with chemotherapy may additionally lead to synergistic effects in HNSCC as other groups have demonstrated for astrocytoma [23]. Despite the lack of MERTK as a prognostic indicator in clinical studies investigating MERTK directed therapies it should be evaluated as a predictive biomarker.

In conclusion, we present the first study comprehensively evaluating the role of MERTK in HNSCC with respect to patient data but also by functional *in-vitro* experiments. Based on these results we suggest that MERTK should be pursued as a therapeutic target in HNSCC.

## MATERIALS AND METHODS

### Immunohistochemical staining

Evaluation of MERTK protein expression was performed on tissue microarrays (TMA) constructed from a cohort of 537 clinically annotated HNSCC patients treated surgically with curative or palliative intent between 1997 and 2011 at the University Hospital Bonn as described previously (Bonn HNSCC cohort) [43]. The study was approved by the institutional review board of the University of Bonn (#148/11). Immunohistochemical staining was conducted as described previously [44]. MERTK antibody was obtained from Sigma-Aldrich (Munich, Germany) (HPA036196) and used in the dilution range of 1:20 to 1:100. CS and SP assessed MERTK protein expression independently for both staining protocols. Staining intensity was classified into four categories: no expression (0), low expression (1), medium expression (2) and high expression (3).

### The cancer genome atlas (TCGA) data

Mutational data for *MERTK* and other potential driver genes (*TP53*, *p16/CDKN2A*, *EGFR*, *NOTCH1* and *PIK3CA*) for TCGA HNSCC patients (n=520) were obtained from cBioportal (www.cbioportal.org, access date 2013-12-10) [45]. The influence of non-synonymous *MERTK* point mutations on protein structure was predicted using the “Mutation Assessor” score provided by cBioportal. Clinical data was downloaded directly from the TCGA Data Portal (https://tcga-data.nci.nih.gov/tcga on 2015-02-08). Level 3 RNA-Seq values normalized for transcript length, isoform abundance and number of MERTK mRNA reads as a fraction of all reads by the RSEM (RNA-Seq by Expectation Maximization) algorithm [46] were downloaded from the TCGA Genome Data Analysis Center (GDAC) Firehose website (https://confluence.broadinstitute.org/display/GDAC/Home)(http://ezid.cdlib.org/id/doi:10.7908/C19P30S6) using the firehose get data-retrieval utility from the 04 February 2015 standard data and analyses run.

### Cell lines

Detroit 562 cells were obtained from Cell Line Service (CLS), Eppelheim, Germany in May 2014 and HN cells were purchased from the Leibnitz Institute DSMZ (Deutsche Sammlung von Mikroorganismen und Zellkulturen), Braunschweig, Germany. Authenticity of HN cells was verified by SNP-Profiling (Multiplexion, Heidelberg, Germany) in January 2014.

Both cell lines were cultured in Dulbecco's Modified Eagle's Medium (DMEM) with L-Glutamine (Gibco® Life technologies, Darmstadt, Germany) supplemented with 10 % fetal bovine serum (FBS) (Biochrom, Berlin, Germany) and 1 % Penicillin Streptomycin (Gibco® Life technologies, Darmstadt, Germany).

### Generation of MERTK overexpressing cells

pDONR223-MERTK was obtained from Addgene, Cambridge, MA, USA (Addgene plasmid # 23900) [47]. MERTK or GFP as control were cloned into the pCSG-IBAwt1 vector (IBA, Göttingen, Germany). Subsequently, HN cells were transfected with either of these vectors using Screenfect A (Genaxxon, Ulm, Germany). Afterwards, cells were constantly cultured with 500 μg/ml Geneticin ®(Life technologies, Darmstadt, Germany) and MERTK overexpression was confirmed by western blot.

### Generation of MERTK knockdown cells

To generate inducible MERTK knockdown cells, two shRNAs against MERTK (TRCN0000000862 and TRCN0000000865) or GFP as control were cloned into the pLKO-Tet-On vector. Afterwards, Detroit 562 cells were lentivirally transduced with these vectors and positive cells were selected using puromycin (Sigma-Aldrich, Munich, Germany). Knockdown was induced using 0.01-1 μg/ml doxycycline (Sigma-Aldrich, Munich, Germany) for 48 to 72 hours. Successful knockdown was confirmed by western blot.

### Proliferation

Proliferation was assessed using Thiazolyl Blue Tetrazolium Bromide (MTT) Assay. 2500 HN cells overexpressing MERTK or GFP were plated in 100 μl medium in 96-Well plates (Corning, Corning, NY, USA). 500 μg/ml MTT (Sigma-Aldrich, Munich, Germany) dissolved in phosphate buffered saline (PBS) (Gibco® Life technologies, Darmstadt, Germany) was added at different time points. Four hours after treatment with MTT 100 μl solubilization buffer (40 % vol/vol Dimethylformamide (Alfa Aesar, Karlsruhe, Germany), 2 % vol/vol glacial acetic (Merck, Darmstadt, Germany), 16 % wt/vol sodium dodecyl sulfate (Applichem, Darmstadt, Germany), pH 4.7) was added and absorbance at 595 nm was measured the next day. For inhibition experiments 5000 Detroit 562 or 2500 HN cells were plated in 50 μl medium before adding different amounts of UNC1062 (AOBIOUS, Gloucester, MA, USA) in 50 μl medium the next day. Knockdown cells were induced for 72 hours with 1 μg/ml doxycycline before plating 5000 cells in 100 μl medium. MTT was added after different time points as described above.

Each experiment was performed in triplicates and repeated at least three times.

### Migration and invasion

For migration and invasion 1-2×10^5^ cells were seeded in 0 or 2 % FBS containing media in the upper chambers of migration (VWR, Darmstadt, Germany) and invasion (VWR, Darmstadt, Germany) inserts. The lower chamber was filled with medium containing 10 % FBS. After 48 hours migrated/invaded cells were fixed with 4 % paraformaldehyde (Merck, Darmstadt, Germany), stained with hemalaun (Waldeck, Münster, Germany) and washed with water. Subsequently ten representative areas of the membrane were counted. For inhibition experiments cells were pretreated with 0.25 μM UNC1062 or the respective amount of DMSO for 24 hours and MERTK knockdown was induced for 48 hours with 0.01 μg/ml doxycycline before performing migration and invasion experiments. Each experiment was repeated at least three times.

### Cell cycle

Cell cycle was analyzed using propidium iodide (PI) staining. Cells were treated with increasing amounts of UNC1062 and were harvested after different time points. Similarly MERTK knockdown cells were harvested 72 hours after induction with doxycycline. Cells were washed two times with sample buffer (0.1 % glucose (Merck, Darmstadt, Germany) in PBS), fixed with ice-cold 70 % ethanol (AppliChem, Darmstadt, Germany) for at least 24 hours at 4°C. Staining was performed with PI staining solution (Sample buffer with 50 μg/ml PI (Sigma-Aldrich, München, Germany) and 50 μg/ml RNAse A (Life technologies, Darmstadt, Germany) for 30 minutes at room temperature before flow cytometry analysis (BD FACSCanto^TM^ II, BD Biosciences, Heidelberg, Germany). Data was evaluated using FlowJo Software (Tree Star, Ashland, OR, USA).

### Apoptosis

Cells were harvested including supernatant and AnnexinV/PI staining was conducted using the Annexin V Apoptosis Detection Kit FITC (eBioscinece, Inc., Diego, CA, USA) according to manufacturers’ instructions. Flow cytometry and data analysis were performed as described above.

### Western blot

For protein extraction cells were lysed for 1 hour on ice with RIPA buffer before centrifuging at 13.000 x g, 4°C for 30 minutes. Protein concentration was measured using Pierce™ BCA Protein Assay Kit (Life technologies, Darmstadt, Germany). Proteins were transferred to a PVDF membrane and blocked with 5 % bovine serum albumin (BSA) or milk in TBS/0.05 % Tween. Incubation with primary antibodies was performed in 5 % BSA or milk in TBS/0.05 % Tween at 4°C overnight. Following primary antibodies were used: AKT (#9272), pAKT (#4060) Erk1/2 (#4695), pERK1/2 (#4370), MERTK (#4319), RhoA (#2117) (Cell Signaling, Danvers, MA, USA), FAK (610088), pFAK (611723) (BD, Biosciences, Heidelberg, Germany), pMERTK (ab192649) (Abcam, Cambridge, UK), beta-actin (A2228) (Sigma-Aldrich, Munich, Germany). After washing with TBS/0.05 % Tween, followed by incubation with HRP-conjugated antibodies against rabbit (#7074) or mouse Ig (#7076) (Cell Signaling, Danvers, MA, USA) in 5 % milk TBS/0.05 % Tween for one hour at room temperature developing was carried out using ECL Western Blotting Reagent (GE Healthcare, Munich, Germany).

### Statistics

Differences of continuous variables between two groups were tested at a two-sided significance level of 0.05 with Students t-Test for normally distributed data or non-parametric Mann-Whitney U test otherwise and the Kruskal-Wallis-H for more than two nominal categories. Significance of monotonic trends in ordinal variables was evaluated with the non-parametric Jonckheere-Terpstra test. P-values for independence of categories in contingency tables was calculated using the Fisher exact test for small to medium sample sizes and Fisher test with 100 000 random Monte Carlo simulations for large sample sizes. Survival was analyzed using Kaplan-Meier estimators with log-rank test and Cox regression models with adjustment for clinical co-variables [48, 49]. All statistical analyses were performed using R software package [50] and IBM SPSS Statistics 22.

## SUPPLEMENTARY TABLES







## Abbreviations

HNSCC: head and neck squamous cell carcinoma
MERTK: MER proto-oncogene tyrosine kinase
FAK: focal adhesion kinase
TCGA: The Cancer Genome Atlas

## References

[1] Jemal A, Siegel R, Xu J, Ward E (2010). Cancer statistics, 2010. CA Cancer J Clin.

[2] Leemans CR, Braakhuis BJ, Brakenhoff RH (2011). The molecular biology of head and neck cancer. Nat Rev Cancer.

[3] Scully C, Bagan JV (2007). Recent advances in Oral Oncology. Oral Oncol.

[4] Mao L, Hong WK, Papadimitrakopoulou VA (2004). Focus on head and neck cancer. Cancer Cell.

[5] Forastiere A, Koch W, Trotti A, Sidransky D (2001). Head and neck cancer. N Engl J Med.

[6] Lemmon MA, Schlessinger J (2010). Cell signaling by receptor tyrosine kinases. Cell.

[7] Temam S, Kawaguchi H, El-Naggar AK, Jelinek J, Tang H, Liu DD, Lang W, Issa JP, Lee JJ, Mao L (2007). Epidermal growth factor receptor copy number alterations correlate with poor clinical outcome in patients with head and neck squamous cancer. J Clin Oncol.

[8] Ang KK, Berkey BA, Tu X, Zhang HZ, Katz R, Hammond EH, Fu KK, Milas L (2002). Impact of epidermal growth factor receptor expression on survival and pattern of relapse in patients with advanced head and neck carcinoma. Cancer Res.

[9] Maurizi M, Almadori G, Ferrandina G, Distefano M, Romanini ME, Cadoni G, Benedetti-Panici P, Paludetti G, Scambia G, Mancuso S (1996). Prognostic significance of epidermal growth factor receptor in laryngeal squamous cell carcinoma. Br J Cancer.

[10] Bonner JA, Harari PM, Giralt J, Azarnia N, Shin DM, Cohen RB, Jones CU, Sur R, Raben D, Jassem J, Ove R, Kies MS, Baselga J, Youssoufian H, Amellal N, Rowinsky EK (2006). Radiotherapy plus cetuximab for squamous-cell carcinoma of the head and neck. N Engl J Med.

[11] Cripps C, Winquist E, Devries MC, Stys-Norman D, Gilbert R (2010). Epidermal growth factor receptor targeted therapy in stages III and IV head and neck cancer. Curr Oncol.

[12] Licitra L, Mesia R, Rivera F, Remenar E, Hitt R, Erfan J, Rottey S, Kawecki A, Zabolotnyy D, Benasso M, Storkel S, Senger S, Stroh C, Vermorken JB (2011). Evaluation of EGFR gene copy number as a predictive biomarker for the efficacy of cetuximab in combination with chemotherapy in the first-line treatment of recurrent and/or metastatic squamous cell carcinoma of the head and neck: EXTREME study. Ann Oncol.

[13] Ang KK, Zhang Q, Rosenthal DI, Nguyen-Tan PF, Sherman EJ, Weber RS, Galvin JM, Bonner JA, Harris J, El-Naggar AK, Gillison ML, Jordan RC, Konski AA, Thorstad WL, Trotti A, Beitler JJ (2014). Randomized phase III trial of concurrent accelerated radiation plus cisplatin with or without cetuximab for stage III to IV head and neck carcinoma: RTOG 0522. J Clin Oncol.

[14] Vermorken JB, Stohlmacher-Williams J, Davidenko I, Licitra L, Winquist E, Villanueva C, Foa P, Rottey S, Skladowski K, Tahara M, Pai VR, Faivre S, Blajman CR, Forastiere AA, Stein BN, Oliner KS (2013). Cisplatin and fluorouracil with or without panitumumab in patients with recurrent or metastatic squamous-cell carcinoma of the head and neck (SPECTRUM): an open-label phase 3 randomised trial. Lancet Oncol.

[15] Martins RG, Parvathaneni U, Bauman JE, Sharma AK, Raez LE, Papagikos MA, Yunus F, Kurland BF, Eaton KD, Liao JJ, Mendez E, Futran N, Wang DX, Chai X, Wallace SG, Austin M (2013). Cisplatin and radiotherapy with or without erlotinib in locally advanced squamous cell carcinoma of the head and neck: a randomized phase II trial. J Clin Oncol.

[16] Study of IMC-A12, Alone or in Combination With Cetuximab, in Patients With Recurrent or Metastatic Squamous Cell Carcinoma (MSCC) of the Head and Neck https://clinicaltrials.gov/ct2/show/NCT00617734.

[17] Comoglio PM, Giordano S, Trusolino L (2008). Drug development of MET inhibitors: targeting oncogene addiction and expedience. Nat Rev Drug Discov.

[18] Elferink LA, Resto VA (2011). Receptor-tyrosine-kinase-targeted therapies for head and neck cancer. J Signal Transduct.

[19] Verma A, Warner SL, Vankayalapati H, Bearss DJ, Sharma S (2011). Targeting Axl and Mer kinases in cancer. Mol Cancer Ther.

[20] Graham DK, Bowman GW, Dawson TL, Stanford WL, Earp HS, Snodgrass HR (1995). Cloning and developmental expression analysis of the murine c-mer tyrosine kinase. Oncogene.

[21] Linger RM, Cohen RA, Cummings CT, Sather S, Migdall-Wilson J, Middleton DH, Lu X, Baron AE, Franklin WA, Merrick DT, Jedlicka P, DeRyckere D, Heasley LE, Graham DK (2013). Mer or Axl receptor tyrosine kinase inhibition promotes apoptosis, blocks growth and enhances chemosensitivity of human non-small cell lung cancer. Oncogene.

[22] Schlegel J, Sambade MJ, Sather S, Moschos SJ, Tan AC, Winges A, DeRyckere D, Carson CC, Trembath DG, Tentler JJ, Eckhardt SG, Kuan PF, Hamilton RL, Duncan LM, Miller CR, Nikolaishvili-Feinberg N (2013). MERTK receptor tyrosine kinase is a therapeutic target in melanoma. J Clin Invest.

[23] Keating AK, Kim GK, Jones AE, Donson AM, Ware K, Mulcahy JM, Salzberg DB, Foreman NK, Liang X, Thorburn A, Graham DK (2010). Inhibition of Mer and Axl receptor tyrosine kinases in astrocytoma cells leads to increased apoptosis and improved chemosensitivity. Mol Cancer Ther.

[24] Yi JH, Jang J, Cho J, Do IG, Hong M, Kim ST, Kim KM, Lee S, Park SH, Park JO, Park YS, Kang WK, Lim HY, Lee J (2015). MerTK is a novel therapeutic target in gastric cancer. Oncotarget.

[25] Wu YM, Robinson DR, Kung HJ (2004). Signal pathways in up-regulation of chemokines by tyrosine kinase MER/NYK in prostate cancer cells. Cancer Res.

[26] Cummings CT, Deryckere D, Earp HS, Graham DK (2013). Molecular pathways: MERTK signaling in cancer. Clin Cancer Res.

[27] Cummings CT, Zhang W, Davies KD, Kirkpatrick GD, Zhang D, DeRyckere D, Wang X, Frye SV, Earp HS, Graham DK (2015). Small Molecule Inhibition of MERTK Is Efficacious in Non-Small Cell Lung Cancer Models Independent of Driver Oncogene Status. Mol Cancer Ther.

[28] Liu J, Zhang W, Stashko MA, Deryckere D, Cummings CT, Hunter D, Yang C, Jayakody CN, Cheng N, Simpson C, Norris-Drouin J, Sather S, Kireev D, Janzen WP, Earp HS, Graham DK (2013). UNC1062, a new and potent Mer inhibitor. Eur J Med Chem.

[29] Stransky N, Egloff AM, Tward AD, Kostic AD, Cibulskis K, Sivachenko A, Kryukov GV, Lawrence MS, Sougnez C, McKenna A, Shefler E, Ramos AH, Stojanov P, Carter SL, Voet D, Cortes ML (2011). The mutational landscape of head and neck squamous cell carcinoma. Science.

[30] Agrawal N, Frederick MJ, Pickering CR, Bettegowda C, Chang K, Li RJ, Fakhry C, Xie TX, Zhang J, Wang J, Zhang N, El-Naggar AK, Jasser SA, Weinstein JN, Trevino L, Drummond JA (2011). Exome sequencing of head and neck squamous cell carcinoma reveals inactivating mutations in NOTCH1. Science.

[31] Kawamata H, Nakashiro K, Uchida D, Harada K, Yoshida H, Sato M (1997). Possible contribution of active MMP2 to lymph-node metastasis and secreted cathepsin L to bone invasion of newly established human oral-squamous-cancer cell lines. Int J Cancer.

[32] Peterson WD, Stulberg CS, Simpson WF (1971). A permanent heteroploid human cell line with type B glucose-6-phosphate dehydrogenase. Proc Soc Exp Biol Med.

[33] Goke F, Franzen A, Hinz TK, Marek LA, Yoon P, Sharma R, Bode M, von Maessenhausen A, Lankat-Buttgereit B, Goke A, Golletz C, Kirsten R, Boehm D, Vogel W, Kleczko EK, Eagles JR (2015). FGFR1 Expression Levels Predict BGJ398 Sensitivity of FGFR1-Dependent Head and Neck Squamous Cell Cancers. Clin Cancer Res.

[34] Wang Y, Moncayo G, Morin P, Xue G, Grzmil M, Lino MM, Clement-Schatlo V, Frank S, Merlo A, Hemmings BA (2013). Mer receptor tyrosine kinase promotes invasion and survival in glioblastoma multiforme. Oncogene.

[35] Nguyen KQ, Tsou WI, Calarese DA, Kimani SG, Singh S, Hsieh S, Liu Y, Lu B, Wu Y, Garforth SJ, Almo SC, Kotenko SV, Birge RB (2014). Overexpression of MERTK receptor tyrosine kinase in epithelial cancer cells drives efferocytosis in a gain-of-function capacity. J Biol Chem.

[36] Guttridge KL, Luft JC, Dawson TL, Kozlowska E, Mahajan NP, Varnum B, Earp HS (2002). Mer receptor tyrosine kinase signaling: prevention of apoptosis and alteration of cytoskeletal architecture without stimulation or proliferation. J Biol Chem.

[37] Knubel KH, Pernu BM, Sufit A, Nelson S, Pierce AM, Keating AK (2014). MerTK inhibition is a novel therapeutic approach for glioblastoma multiforme. Oncotarget.

[38] Rogers AE, Le JP, Sather S, Pernu BM, Graham DK, Pierce AM, Keating AK (2012). Mer receptor tyrosine kinase inhibition impedes glioblastoma multiforme migration and alters cellular morphology. Oncogene.

[39] Abraham MT, Kuriakose MA, Sacks PG, Yee H, Chiriboga L, Bearer EL, Delacure MD (2001). Motility-related proteins as markers for head and neck squamous cell cancer. Laryngoscope.

[40] Bourguignon LY, Gilad E, Brightman A, Diedrich F, Singleton P (2006). Hyaluronan-CD44 interaction with leukemia-associated RhoGEF and epidermal growth factor receptor promotes Rho/Ras co-activation, phospholipase C epsilon-Ca2+ signaling, and cytoskeleton modification in head and neck squamous cell carcinoma cells. J Biol Chem.

[41] Smith LP, Thomas GR (2006). Animal models for the study of squamous cell carcinoma of the upper aerodigestive tract: a historical perspective with review of their utility and limitations. Part A. Chemically-induced de novo cancer, syngeneic animal models of HNSCC, animal models of transplanted xenogeneic human tumors. Int J Cancer.

[42] Zhang W, DeRyckere D, Hunter D, Liu J, Stashko MA, Minson KA, Cummings CT, Lee M, Glaros TG, Newton DL, Sather S, Zhang D, Kireev D, Janzen WP, Earp HS, Graham DK (2014). UNC2025, a potent and orally bioavailable MER/FLT3 dual inhibitor. J Med Chem.

[43] Goke F, Bode M, Franzen A, Kirsten R, Goltz D, Goke A, Sharma R, Boehm D, Vogel W, Wagner P, Lengerke C, Kristiansen G, Kirfel J, Van Bremen T, Bootz F, Heasley LE (2013). Fibroblast growth factor receptor 1 amplification is a common event in squamous cell carcinoma of the head and neck. Mod Pathol.

[44] Wilbertz T, Wagner P, Petersen K, Stiedl AC, Scheble VJ, Maier S, Reischl M, Mikut R, Altorki NK, Moch H, Fend F, Staebler A, Bass AJ, Meyerson M, Rubin MA, Soltermann A (2011). SOX2 gene amplification and protein overexpression are associated with better outcome in squamous cell lung cancer. Mod Pathol.

[45] Cerami E, Gao J, Dogrusoz U, Gross BE, Sumer SO, Aksoy BA, Jacobsen A, Byrne CJ, Heuer ML, Larsson E, Antipin Y, Reva B, Goldberg AP, Sander C, Schultz N (2012). The cBio cancer genomics portal: an open platform for exploring multidimensional cancer genomics data. Cancer Discov.

[46] Li B, Ruotti V, Stewart RM, Thomson JA, Dewey CN (2010). RNA-Seq gene expression estimation with read mapping uncertainty. Bioinformatics.

[47] Johannessen CM, Boehm JS, Kim SY, Thomas SR, Wardwell L, Johnson LA, Emery CM, Stransky N, Cogdill AP, Barretina J, Caponigro G, Hieronymus H, Murray RR, Salehi-Ashtiani K, Hill DE, Vidal M (2010). COT drives resistance to RAF inhibition through MAP kinase pathway reactivation. Nature.

[48] Kaplan E, Meier P (1958). Nonparametric Estimation from Incomplete Observations. J Am Stat Assoc.

[49] Cox D (1972). Regression models and life tables. J R Stat Soc Ser B.

[50] R RDCT (2011). R: A Language and Enviroment for Statistical Computing. R Found Stat Comput.

