# Supplementary material for: MERTK as a novel therapeutic target in head and neck cancer
Source: Oncotarget. 2016 Apr 13;7(22):32678–94. doi: 10.18632/oncotarget.8724 (PMC5078043; doi:10.18632/oncotarget.8724)
Supplement: Supplementary file 1 [file oncotarget-07-32678-s001.pdf]

MERTK as a novel therapeutic target in head and neck cancer

SUPPLEMENTARY TABLES

Supplementary Table S1: MERTK expression in the Bonn HNSCC sub-cohorts 1 and 2

**Sub-cohort 1**						
	**total number of patients n=537**	**MERTK negative**	**low MERTK**	**medium MERTK**	**high MERTK**	**p-value**
**Tissues Available**						
Normal	10 (10*)	6 (60.0 %)	4 (40.0 %)	0	0	normal vs. tumor tissue 0.042
Primary tumor	307 (15*)	84 (27.4 %)	106 (34.5 %)	88 (28.7 %)	29 (9.4 %)	
Lymph node metastasis	29 (4*)	13 (44.8 %)	7 (24.1 %)	9 (31.1 %)	0	
local recurrence	4 (1*)	2 (50.0 %)	1 (25.0 %)	1 (25.0 %)	0	
**Sub-cohort 2**	total number of patients n=537	MERTK negative	low MERTK	medium MERTK	high MERTK	
**Tissues Available**						
Normal	21 (9*)	15 (71.4 %)	6 (28.6 %)	0	0	normal vs. tumor tissue 0.010
Primary tumor	154 (37*)	55 (35.7 %)	56 (36.4 %)	23 (14.9 %)	20 (13.0 %)	
Lymph node metastasis	164 (14*)	66 (40.3 %)	45 (27.4 %)	40 (24.4 %)	13 (7.9 %)	
local recurrence	50 (7*)	25 (50.0 %)	11 (22.0 %)	11 (22.0 %)	3 (6.0 %)	

Summary of available tissue of the Bonn HNSCC sub-cohorts used for MERTK expression analyses. For some patients tissue for more than one entity (e.g. normal and primary tumor) could be used in IHC (* number of stained tissue samples for which clinical information was not available). Significance was tested with Fisher test: Monte Carlo, 100 000 random samples.

Supplementary Table S2: Clinico-pathological features of the Bonn HNSCC sub-cohorts 1 and 2. Summary of clinico-pathological features of the sub-cohorts used for MERTK expression analyses (SD, standard deviation). Significance was tested with (1) Fisher test: exact, (2) Fisher test: Monte Carlo, 100 000 random samples.

See Supplementary File 1

Supplementary Table S3: Cox Model for the Bonn HNSCC cohort

**Cox Model for the Bonn HNSCC cohort (Overall Model p-value < 0.0001)**				
**Co-variable**		**Hazard ratio**	**95 % Conf. Interval**	**p-value**
MERTK	[medium or high vs. negative or low]	1.215	0.823 – 1.793	0.327
Alcohol	[occasional vs. non-drinker]	0.897	0.511 – 1.575	0.705
	[medium-heavy vs. non-drinker]	1.865	1.156 – 3.008	**0.011**
Tobacco	[ever-smoker vs. never-smoker]	0.943	0.488 – 1.822	0.860
HPV status	[positive]	0.359	0.143 – 0.904	**0.030**
Age	[years]	1.030	1.009 – 1.051	**0.005**
Tumor stage	[II vs. I]	1.699	0.784 – 3.682	0.180
	[III vs. I]	2.362	1.168 – 4.779	**0.017**
	[IV vs. I]	3.178	1.718 – 5.877	**<0.0003**

Multivariable Cox models evaluating effects of MERTK expression for patients with medium or high MERTK expression (High MERTK) adjusted for clinical parameters known to be associated with prognosis in HNSCC.

Supplementary Table S4: MERTK expression and clinico-pathological features of the TCGA HNSCC cohort. Summary of clinico-pathological features of the TCGA HNSCC cohort. MERTK mRNA expression was determined by RNA-Seq as RSEM and log2-transformed for analyses. Differences were tested with (1) Mann-Whitney-U Test for dichotome, (2) Kruskal-Wallis test for nominal and (3) Jonckheere-Terpstra test for monotonic trend for ordinal categories. All tests were done two-sided (SD, standard deviation).

See Supplementary File 2
