# Supplementary material for: MERTK as a novel therapeutic target in head and neck cancer
Source: Oncotarget. 2016 Apr 13;7(22):32678–94. doi: 10.18632/oncotarget.8724 (PMC5078043; doi:10.18632/oncotarget.8724)
Supplement: Supplementary file 2 [file oncotarget-07-32678-s002.docx]

**Supplementary Table S2**

**Clinico-pathological features of the Bonn HNSCC sub-cohorts 1 and 2.**

|  | **Sub-cohort 1**  patients with clinical data and primary tumors n=292 | **Sub-cohort 2**  patients with clinical data and primary tumors  n=117 | p-value  (difference between cohorts) |
| --- | --- | --- | --- |
| **Gender** |  |  |  |
| female | 80 (27.4 %) | 19 (16.2 %) | 0.021 ^(1)^ |
| male | 212 (72.6 %) | 98 (83.8 %) |  |
| **Age [years, SD]** | 62.68 [11.2] | 61.24 [10.1] |  |
| **Anatomic localization of Primary** |  |  |  |
| Oral Cavity | 87 (29.8 %) | 12 (10.2 %) | <0.0001 ^(2)^ |
| Oropharynx | 112 (38.4 %) | 20 (17.1 %) |  |
| Hypopharynx | 28 (9.6 %) | 25 (21.4 %) |  |
| Larynx | 61 (20.8 %) | 58 (49.6 %) |  |
| Unknown | 4 (1.4 %) | 2 (1.7 %) |  |
| **Tobacco** |  |  |  |
| Never-Smoker | 31 (10.6 %) | 4 (3.4 %) | 0.002 ^(1)^ |
| Ever-Smoker | 178 (61.0 %) | 100 (85.5 %) |  |
| Unknown | 83 (28.4 %) | 13 (11.1 %) |  |
| **Alcohol** |  |  |  |
| Non-drinker | 79 (27.1 %) | 28 (23.9%) | 0.074 ^(2)^ |
| Occasional | 41 (14.0 %) | 30 (25.6 %) |  |
| Medium-Heavy | 80 (27.4 %) | 36 (30.8 %) |  |
| Unknown | 92 (31.5 %) | 23 (19.7 %) |  |
| **HPV Status** |  |  |  |
| Positive | 29 (9.9 %) | 6 (5.1 %) | 0.170 ^(1)^ |
| Negative | 263 (90.1 %) | 111 (94.9 %) |  |
| **T-Stage of Primary** |  |  |  |
| T1 | 78 (26.7 %) | 23 (19.7 %) | 0.576 ^(2)^ |
| T2 | 96 (32.9 %) | 40 (34.2 %) |  |
| T3 | 66 (22.6 %) | 31 (26.5 %) |  |
| T4 | 49 (16.8 %) | 22 (18.8 %) |  |
| Unkown | 3 (1.0 %) | 1 (0.8 %) |  |
| **N Stage of Primary** |  |  |  |
| N0 | 138 (47.3 %) | 47 (40.2 %) | 0.278 ^(2)^ |
| N1 | 45 (15.4 %) | 22 (18.8 %) |  |
| N2 | 97 (33.2 %) | 46 (39.3 %) |  |
| N3 | 5 (1.7 %) | 0 (0.0 %) |  |
| Unkown | 7 (2.4 %) | 2 (1.7 %) |  |
| **M Stage of Primary** |  |  |  |
| M0 | 281 (96.2 %) | 114 (97.4 %) | 0.765 ^(1)^ |
| M1 | 10 (3.4 %) | 3 (2.6 %) |  |
| Unknown | 1 (0.4 %) | 0 (0.0 %) |  |
| **Tumor stage of Primary** |  |  |  |
| I | 58 (19.9 %) | 18 (15.4 %) | 0.815 ^(2)^ |
| II | 41 (14.0 %) | 17 (14.5 %) |  |
| III | 59 (20.2 %) | 23 (19.7 %) |  |
| IV | 127 (43.5 %) | 57 (48.7 %) |  |
| Unknown | 7 (2.4 %) | 2 (1.7 %) |  |
| **Grading** |  |  |  |
| G1 | 2 (0.7 %) | 0 (0.0 %) | 0.138 ^(2)^ |
| G2 | 14 (4.8 %) | 1 (0.8 %) |  |
| G3 | 156 (53.4 %) | 61 (52.1 %) |  |
| G4 | 69 (23.6 %) | 34 (29.2 %) |  |
| Unknown | 51 (17.5 %) | 21 (17.9 %) |  |

Summary of clinico-pathological features of the sub-cohorts used for MERTK expression analyses (SD, standard deviation). Significance was tested with (1) Fisher test: exact, (2) Fisher test: Monte Carlo, 100 000 random samples.
