# Supplementary material for: MERTK as a novel therapeutic target in head and neck cancer
Source: Oncotarget. 2016 Apr 13;7(22):32678–94. doi: 10.18632/oncotarget.8724 (PMC5078043; doi:10.18632/oncotarget.8724)
Supplement: Supplementary file 3 [file oncotarget-07-32678-s003.docx]

**Supplementary Table S4**

**MERTK expression and clinico-pathological features of the TCGA HNSCC cohort.**

|  | **TCGA HNSCC cohort**  (Number of patients n=520) | **MERTK mRNA Expression log2 (RSEM)**  Mean (SD) | **p-value** |
| --- | --- | --- | --- |
| **Tissues Available** | | | |
| Normal Mucosa | 44 | 6.92 (1.10) | p = 0.16 ^(1)^ |
| Primary Tumor | 520 | 7.16 (1.43) |  |
| **Gender** | | | |
| Male | 361 (69.4 %) | 7.03 (1.34) | p = 0.34 ^(1)^ |
| Female | 130 (25.0 %) | 7.18 (1.48) |  |
| Unknown | 29 (5.6 %) | 7.08 (1.37) |  |
| **Age [years, SD]** | 61.3 (11.9) |  |  |
| **Anatomic localization of**  **Primary** | | | |
| Oral Cavity | 299 (57.5 %) | **6.80 (1.29)** | **p < 0.001** ^(2)^ |
| Oropharynx | 72 (13.8 %) | **7.70 (1.37)** |  |
| Hypopharynx/  Larynx | 120 (23.1 %) | **7.65 (1.59)** |  |
| Unknown | 29 (5.6 %) | 7.54 (1.30) |  |
| **Tobacco** | | | |
| Never-Smoker | 106 (20.4 %) | **6.86 (1.44)** | **p = 0.034** ^(1)^ |
| Ever-Smoker | 372 (71.5 %) | **7.24 (1.39)** |  |
| Unknown | 42 (8.1 %) | 7.23 (1.74) |  |
| **Alcohol** | | | |
| Non-drinker | 48 (9.2 %) | 6.94 (1.32) | p = 0.47 ^(3)^ |
| Occasional | 36 (6.9 %) | 7.21 (1.07) |  |
| Medium-Heavy | 126 (24.2 %) | 7.16 (1.46) |  |
| Unknown | 310 (59.6 %) | 7.19 (1.48) |  |
| **HPV Status** | | | |
| Positive | 68 (13.1 %) | **7.96 (1.31)** | **p < 0.001** ^(1)^ |
| Negative | 423 (81.3 %) | **6.97 (1.32)** |  |
| Unknown | 29 (5.6 %) | 7.54 (1.30) |  |
| **T-Stage of Primary** | | | |
| T1 | 46 (8.8 %) | 6.98 (1.02) | p = 0.92 ^(3)^ |
| T2 | 148 (28.5 %) | 7.15 (1.60) |  |
| T3 | 117 (22.5 %) | 6.92 (1.39) |  |
| T4 | 179 (34.4 %) | 7.23 (1.21) |  |
| Unknown | 30 (5.8 %) | 7.57 (1.29) |  |
| **N Stage of Primary** | | | |
| N0 | 214 (41.2 %) | **6.95 (1.22)** | **p = 0.011** ^(3)^ |
| N1 | 69 (13.3 %) | **7.22 (1.53)** |  |
| N2 | 194 (37.3 %) | **7.22 (1.44)** |  |
| N3 | 10 (1.9 %) | **8.10 (1.38)** |  |
| Unknown | 33 (6.3 %) | 7.46 (1.26) |  |
| **M Stage of Primary** | | | |
| M0 | 468 (90.0 %) | 7.16 (1.39) | p = 0.33 ^(1)^ |
| M1 | 4 (0.8 %) | 6.49 (0.33) |  |
| Unknown | 48 (9.2 %) | 7.30 (1.25) |  |
| **Grading** | | | |
| G1 | 60 (11.5 %) | **6.79 (0.859** | **p < 0.001**^(3)^ |
| G2 | 292 (56.2 %) | **6.97 (1.37)** |  |
| G3 | 114 (21.9 %) | **7.34 (1.45)** |  |
| G4 | 6 (1.2 %) | **8.27 (1.08)** |  |
| Unknown | 48 (9.2 %) | 7.63 (1.40) |  |
| **Tumor stage (clinical)** | | | |
| I | 19 (3.7 %) | **6.35 (0.91)** | **p = 0.046** ^(3)^ |
| II | 98 (18.8 %) | **6.75 (1.42)** |  |
| III | 97 (18.7 %) | **6.96 (1.45)** |  |
| IV | 264 (50.8 %) | **7.39 (1.31)** |  |
| Unknown | 42 (8.1 %) | 7.21 (1.31) |  |

Summary of clinico-pathological features of the TCGA HNSCC cohort. MERTK mRNA expression was determined by RNA-Seq as RSEM and log2-transformed for analyses. Differences were tested with (1) Mann-Whitney-U Test for dichotome, (2) Kruskal-Wallis test for nominal and (3) Jonckheere-Terpstra test for monotonic trend for ordinal categories. All tests were done two-sided (SD, standard deviation).
